# Étude de faisabilité du dispositif « Passerelle » couplant transfert monétaire non fléché et orientation sociale destiné à des ménages en situation de précarité en France

**DOI:** 10.17269/s41997-025-01120-7

**Published:** 2026-04-29

**Authors:** Pierre Gallinari-Safar, Sabrina Eymard-Duvernay, Christophe Dubois, Nicole Darmon, Maria Somaraki, Massimo Hulot, Clara Souchereau, Emilie Martin, Emmanuel Ollivier, Hélène Quéau, Vigdis Gosset, Marlène Perignon

**Affiliations:** 1 MoISA, Univ Montpellier, CIHEAM-IAMM, CIRAD, INRAE, Institut Agro, IRD, Montpellier, France; 2 ActAP, Marseille, France; 3 Action Contre la Faim, Montreuil, France; 4 Fondation de L’Armée du Salut, Paris, France

**Keywords:** Insécurité alimentaire, Précarité, Transfert monétaire, Accès aux droits, Inégalités sociales de santé, Intervention, Food insecurity, Poverty, Cash transfer, Access to rights, Social inequalities in health, Intervention

## Abstract

**Intervention:**

The new “Passerelle” program aims to reduce socioeconomic inequalities in diet in France, by offering an untargeted cash transfer (CT) coupled with social guidance to households in precarious situations.

**Objectives:**

Assess the feasibility of the program and data collection for monitoring indicators, to inform the design of a future trial to evaluate its effectiveness.

**Methods:**

Single-group pre-post-test feasibility study. The program was implemented in Montreuil (France) with households in precarious situations identified by local social actors. The feasibility of the program was assessed in terms of implementation of household targeting (number and socio-economic characteristics of participants), intervention components (participation rates), and user acceptability (questionnaire and qualitative survey). The feasibility of data collection was assessed by participation in surveys before inclusion (t0) and after 3 (t1) and 6 months (t2), response rates and responsiveness of food security and well-being indicators.

**Results:**

Two hundred households took part in the program, 90% were in monetary poverty, 82% in food insufficiency, 97% received the cash transfer card, 99% attended the social orientation meeting, over 95% said they were satisfied with the program, and 69% said they improved their food purchases. The qualitative survey documented the reasons for acceptability. Participation rates at t0, t1 and t2 were 93%, 76% and 73%. Non-response rates were below 5% for most indicators. Follow-up indicators ranged from + 19% to + 99% at t1.

**Conclusion:**

This study demonstrates the feasibility of the program and collecting data from the target population to monitor food security and well-being indicators. It confirms the interest of a future trial to evaluate the program effectiveness, in order to contribute to reflections on policies to combat food insecurity in France. An analysis of the program theory, explaining the expected changes, mechanisms and conditions of impact would be beneficial for future research.

**Supplementary Information:**

The online version contains supplementary material available at 10.17269/s41997-025-01120-7.

## Résumé

**Intervention:**

Le nouveau dispositif « Passerelle » vise à réduire les inégalités sociales liées à l’alimentation en France en proposant un transfert monétaire (TM) non fléché et une orientation sociale à des ménages en situation de précarité.

**Objectifs:**

Evaluer la faisabilité du dispositif et de la collecte de données pour le suivi d’indicateurs afin d’informer la conception d’un futur essai pour évaluer son efficacité.

**Méthodes:**

Etude de faisabilité de type pré-post test à groupe unique. Le dispositif a été déployé à Montreuil (France) auprès de ménages en situation de précarité identifiés par des acteurs sociaux de proximité. La faisabilité du dispositif est évaluée en matière de mise en œuvre du ciblage des ménages (nombre et caractéristiques socio-économiques des participants), des volets de l’intervention (taux de participation), et d’acceptabilité des usagers (questionnaire et enquête qualitative). La faisabilité de la collecte de données est évaluée par la participation aux enquêtes avant inclusion (t0) et après 3 (t1) et 6 mois (t2), les taux de réponses et la réactivité d’indicateurs de sécurité alimentaire et de bien-être.

**Résultats:**

Deux cent ménages ont participé au dispositif, 90% était en situation de pauvreté monétaire, 82% en insuffisance alimentaire, 97% ont reçu la carte de TM, 99% ont participé à l’orientation sociale, plus de 95% déclaraient être satisfait du dispositif, 69% déclaraient avoir pu améliorer leurs achats alimentaires. L’enquête qualitative a documenté les raisons d’acceptabilité. Les taux de participation à t0, t1 et t2 étaient de 93%, 76% et 73%. Les taux de non-réponse étaient < 5% pour la majorité des indicateurs. Les indicateurs de suivi ont varié de + 19% à + 99% à t1.

**Conclusion:**

Cette étude montre la faisabilité du dispositif et de la collecte de données auprès de la population cible pour le suivi d’indicateurs de sécurité alimentaire et de bien-être. Elle confirme l’intérêt d’un futur essai pour évaluer l’efficacité du dispositif afin d’alimenter les réflexions sur les politiques de lutte contre la précarité alimentaire en France. Une analyse de la théorie du programme explicitant les changements attendus, les mécanismes et les conditions d’impact serait bénéfique pour de futures recherches.

## Introduction

La prévalence d’insécurité alimentaire en France a été estimée à 8 millions de personnes en 2015 (AnSES, 2017). Dans la période récente, un ensemble d’indicateurs laissent présager d’une intensification et d’une hausse de la prévalence des situations d’insécurité alimentaire sur le territoire: précarisation et hausse des effectifs des publics de l’aide alimentaire (Accardo et al., 2022a, 2022b), apparition de nouveaux profils au sein de leurs files actives (Le Méner et al., 2020), niveau de restriction alimentaire important chez les étudiants (Guénée et al., 2022) et les ménages en situation de pauvreté monétaire (Secours Populaire et IPSOS, 2024), hausse des prix des produits alimentaires (INSEE, 2023). Les crises sanitaire et économique récentes cristallisent les limites d’un système d’aide alimentaire reposant essentiellement sur des distributions de denrées organisées par des acteurs associatifs, rendant saillante l’incapacité de ce modèle à répondre de manière satisfaisante et sur le long terme aux besoins d’une part croissante de la population (Caillavet et al., 2021; Darmon et al., 2020). Repenser la lutte contre la précarité alimentaire en France est donc devenu un enjeu majeur; il est urgent d’identifier, expérimenter et évaluer l’efficacité de dispositifs de prévention de l’insécurité alimentaire qui répondent aux besoins des populations (Arnold et al., 2011).

Des évaluations portant sur des interventions de transfert monétaire (TM), qui consistent en la distribution directe d’un moyen de paiement, ont généré dans les pays à faibles revenus de nombreuses preuves de leurs effets positifs sur la santé physique, mentale, l’insécurité, et l’éducation des usagers (Arnold et al., 2011; Manley et al., 2012). Si dans ce contexte se sont essentiellement généralisées des évaluations portant sur des TM associés à des conditions de réception ou des restrictions d’usage, des études plus récentes ont montré que les TM inconditionnels ont également des impacts positifs sur l’insécurité alimentaire, le bien-être, la santé physique et mentale des ménages (Bastagli et al., 2016; Dwyer et al., 2022; Haushofer et Shapiro, 2016), tout en étant susceptibles de diminuer les coûts associés à la mise en place et la gestion des restrictions, ainsi que de réduire les difficultés d’accès, la stigmatisation et le non-recours (Yoshino et al., 2023). Dans les pays à revenu élevé, peu d’études évaluent l’impact des TM sur la santé, et plus particulièrement sur l’insécurité alimentaire. Celles qui le font sont essentiellement des évaluations portant sur des dispositifs de TM gouvernementaux dont l’usage est restreint à la réalisation de dépenses alimentaires. Leur efficacité sur l’insécurité alimentaire et la santé (Bitler et Seifoddini, 2019; Boccia et al., 2023; Carlson et LLobrera, 2022; Mabli et Ohls, 2015) fait aujourd’hui consensus, mais les restrictions d’usage à l’alimentation sont susceptibles de reconduire les problèmes d’accès, d’acceptabilité et de non-recours (Johnson, s. d.; Liu et Liu, 2016; Panzera et al., 2017) ainsi que d’entraver la liberté des ménages à répondre à des besoins essentiels hors alimentation.

En France, à ce jour, seules 2 études expérimentales ont évalué l’effet de TM. Il s’agissait de la délivrance de chèques alimentaires fléchés vers l’achat de fruits et légumes et les résultats montrent une réduction de la proportion de petits consommateurs de fruits et légumes, pour l’une d’elle uniquement chez les enfants (Bihan et al., 2012; Buscail et al., 2018, 2019). Si les TM ciblés vers les fruits et légumes ont fait la preuve en contexte de pays du Nord qu’ils tendent à modifier de manière modérée les comportements de consommation en levant les barrières d’accès à certains produits (An, 2013), leur effet à long-terme sont incertains, et les format et restrictions d’usage ne permettent pas de répondre aux problèmes d’acceptabilité, d’autonomie, et de non-recours précédemment évoqués (Brocard et Saujot, 2022; Buscail et al., 2018). Il existe encore peu d’études dans des pays à revenu élevé ayant évalué l’acceptabilité et les effets de dispositifs de TM non fléchés sur l’insécurité alimentaire et la santé.

Le nouveau dispositif « Passerelle» développé par deux associations en France (Action Contre la Faim en partenariat avec la Fondation de l’Armée du Salut) visait à réduire les inégalités sociales en matière d’alimentation en proposant à des ménages en situation de précarité un TM non fléché couplé à une orientation sociale. Plusieurs éléments amènent à considérer le dispositif « Passerelle» comme une intervention complexe, nécessitant de mobiliser une approche méthodologique spécifique pour évaluer ses effets. D’une part, le dispositif comprend différentes composantes interventionnelles (un TM et une orientation sociale) pouvant interagir entre elles et dont les effets sont susceptibles de dépendre du contexte dans lequel elles s’insèrent. D’autre part, il mobilise différents groupes d’acteurs (centres sociaux, associations, établissements publics, personnes en situation de précarité), et vise à agir sur différents déterminants de l’insécurité alimentaire: l’accessibilité financière à l’alimentation et l’accès aux droits sociaux. L’étude présentée dans cet article s’appuie sur le cadre du Medical Research Council (MRC) pour le développement d'interventions complexes (Richards et Hallberg, 2015; Skivington et al., 2021) qui identifie la faisabilité comme une des phases clé des recherches sur les interventions complexes avant d’entreprendre l’évaluation de son efficacité. Cette phase préconise d’explorer la faisabilité et l’acceptabilité de l’intervention elle-même mais également du processus de collecte de données pour son évaluation afin de déterminer l’intérêt et la faisabilité d'un éventuel essai randomisé de plus grande ampleur (Eldridge et al., 2016).

Ainsi, l’objectif principal de cette étude était d’évaluer la faisabilité du dispositif « Passerelle », plus spécifiquement la faisabilité de mise en œuvre des 2 volets du dispositif (TM et orientation sociale) et du ciblage des ménages en situation de précarité par les acteurs sociaux de proximité, ainsi que son acceptabilité par les usagers. L’objectif secondaire était d’évaluer la faisabilité de la procédure de collecte de données pour le suivi d’indicateurs de sécurité alimentaire et de bien-être et obtenir des données préliminaires sur le niveau d’insécurité alimentaire de la population ciblée. L’objectif général de cette étude de faisabilité était d’informer la conception d’un futur essai randomisé contrôlé pour évaluer l’efficacité du dispositif.

## Méthodes

### Design de l’étude

Il s’agit d’une étude de faisabilité de type pré-test post-test à groupe unique. Le dispositif « Passerelle» a été déployé en 2022 auprès de ménages en situation de précarité dans cinq quartiers classés prioritaires de la ville (QPV) de Montreuil, France. Le déploiement du dispositif s’est organisé en deux phases de 4 mois: de janvier à avril (phase 1), et de juin à septembre (phase 2). La collecte de données comprenait une enquête qualitative par entretiens semi-directifs avec des ménages ayant participé au dispositif, et une enquête quantitative par questionnaire administrés auprès de l’ensemble des ménages participants avant (t0), trois mois (t1) et six mois (t2) après l’entrée dans le dispositif, pour chacune des deux phases de déploiement. Les questionnaires ont été administrés en face à face dans les centres sociaux de Montreuil.

La taille d’échantillon de cette étude de faisabilité était destinée à pouvoir estimer avec une précision suffisante la proportion de sécurité alimentaire nécessaire à la conception du futur essai randomisé contrôlé visant à évaluer l’efficacité du dispositif: un échantillon de 200 ménages permettra d’estimer les proportions de sécurité alimentaire avec une précision entre 4% (± 0.04) et 7% (± 0.07) pour des proportions entre 10%(/90%) et 50% pour un niveau de confiance de 95%.

Le protocole de l’étude a obtenu un avis favorable du Comité d’Evaluation Ethique de l’Inserm (IRB00003888, avis n°22–890). Tous les individus représentants les ménages participants ont consenti par écrit à participer à l’étude.

### Intervention

La description détaillée de l’intervention (le dispositif « Passerelle ») selon la grille TIDieR-PHP (Campbell et al., 2018) est fournie dans le Tableau Supplémentaire 1. Le dispositif « Passerelle » est un nouveau dispositif de lutte contre la précarité alimentaire proposé par 2 associations en France: Action Contre la Faim et la Fondation de l’Armée du Salut. L’objectif du dispositif est de réduire les inégalités sociales liées à l’alimentation en agissant sur des déterminants sociaux: l’accessibilité financière des ménages en situation de précarité et leur accès aux droits sociaux. Le développement de ce nouveau dispositif repose sur différents constats: les modalités de l’aide alimentaire conventionnelle en France—majoritairement distribuée sous forme de denrées—limitent la liberté de choix des usagers, le respect de la dignité des personnes et le recours à cette aide (Caillavet et al., 2021); plus largement le non-recours aux droits s’impose comme une problématique prioritaire et appelle à repenser l’accessibilité des droits et des services (CNCDH, 2022).

Le dispositif « Passerelle» est proposé à des ménages en situation de précarité et comprend 2 volets: un TM non fléché et une orientation sociale individualisée. Le volet TM consiste à distribuer une allocation non fléchée de 63€/mois par personne du ménage pendant 4 mois sous forme de carte de paiement. Ce montant a été défini sur la base du coût minimum nécessaire pour respecter l’ensemble des recommandations nutritionnelles (estimé à 3,85 €/j) (Maillot et al., 2017) et avec l’intention de couvrir les besoins au-delà du strict minimum alimentaire (e.g. transport) et de prendre en compte l’inflation observée en 2022, soit un montant réévalué à 4,5€/jour/personne, pour deux semaines par mois. Deux types de carte ont été expérimentés pour la mise à disposition du TM: i) carte « Nickel», utilisable dans tous types de magasins et permettant aussi le retrait d’espèces et les paiements en ligne, et ii) carte « Cohésia», utilisable dans toutes les enseignes d’alimentation (supermarché, restaurant, boulangerie, boucherie, épicerie,…), de santé (pharmacie, médecin, hôpital), d’énergie (station-essence), de transports (taxi, bus, métro, train) et de services (blanchisserie, poste, impôts). Lorsque les pièces d’identité nécessaires à la création des cartes n’étaient pas transmises à temps ou n’étaient pas valides, le TM a été distribué sous forme de chèques services. Le volet TM (distribution de la carte et réalisation des versements mensuels) a été mis en œuvre par le personnel d'Action Contre la Faim.

Le volet « orientation sociale» du dispositif consiste à proposer aux participants un entretien individuel facultatif avec un travailleur social afin d’établir un diagnostic social individualisé. En fonction du diagnostic, les ménages étaient alors orientés vers des structures associatives présentes sur le territoire (épicerie solidaire, association d’accompagnement psychologique gratuit, écrivain public, etc.) ou de l’accès aux droits (intermédiation d’échelonnement de dette auprès de l’OPHM, formations via Pole Emploi, accès à des chantiers d’insertion etc.). Les entretiens d'orientation sociale ont été menés dans les centres sociaux de Montreuil par des coordinateur.rices sociaux.ales de la Fondation de l'Armée du Salut. Un travail de cartographie des dispositifs et de rencontres des acteurs de l’action sociale présents sur le territoire montreuillois a été réalisé par les coordinateur.rices sociaux.ales en amont des rendez-vous avec les participants afin de proposer des orientations adéquates.

L’identification des ménages en situation de précarité à qui le dispositif était proposé a été réalisé dans une approche « d’aller vers» par les acteurs sociaux des QPV (centres sociaux, antennes de quartier et associations de proximité). Les ménages devaient être en situation administrative régulière et de logement ordinaire. Une liste de critères indicatifs pour l’identification de ménages en situation de précarité a été transmise aux acteurs sociaux, basée sur une approche large de la précarité recoupant des dimensions à la fois d’ordre socioéconomique (faibles revenus, situation professionnelle instable ou précaire), familial (foyer monoparentaux, personnes isolées) ou administratif (rupture de droit ou situation de non-recours). Aucune pièce justificative n’était exigée au cours du processus de recrutement, mis à part une photocopie d’une pièce d’identité qui était nécessaire pour créer la carte de TM.

### Evaluation de la faisabilité du dispositif

L’évaluation de la faisabilité du dispositif « Passerelle» portait sur la mise en œuvre de l’identification des ménages en précarité par les acteurs sociaux de proximité, la mise en œuvre des 2 volets du dispositif, et l’acceptabilité perçue par les usagers.

La faisabilité du recrutement des ménages en situation de précarité a été évaluée par le nombre d’acteurs sociaux ayant contribué à cette étape de ciblage, le nombre de ménage participant au dispositif, et leur caractéristiques socio-économiques. Ces dernières ont été collectées lors de l’enquête à t0 qui comprenait des questions relatives à l’âge, au sexe et au statut vis à vis de l’emploi de l’individu représentant le ménage participant, ainsi qu’au revenu, à la composition et à l’endettement du ménage. Lors de la phase 2, l’enquête t0 incluait également des questions permettant le calcul de l’indice de privationsmatérielle et sociale (IPMS). Les ménages étaient considérés en situation de pauvreté monétaire lorsque leur niveau de vie était inférieur au seuil de pauvreté fixé à 60% du niveau de vie médian; en situation de pauvreté en conditions de vie lorsque lorsqu’ils cumulaient au moins 5 privations ou difficultés matérielles parmi les 13 de la liste de l’IPMS; en situation de grande pauvreté lorsqu’ils cumulaient pauvreté monétaire sévère (en dessous du seuil de pauvreté à 50%) et pauvreté en conditions de vie sévère (au moins 7 privations sur 13).

La faisabilité de la mise en œuvre des 2 volets a été évaluée par le nombre de carte de TM distribuées, et le nombre d’entretiens d’orientation sociale réalisés.

L'acceptabilité est un concept à multiples facettes qui reflète la mesure dans laquelle les personnes recevant une intervention la considèrent comme appropriée, sur la base des réponses cognitives et émotionnelles anticipées ou expérimentées à l'égard de l'intervention (Sekhon et al., 2017). Dans cette étude, l’acceptabilité a été étudiée de manière rétrospective en interrogeant les personnes après qu’elles aient expérimenté le dispositif. L’ensemble des participants ont été interrogés par questionnaire sur leur niveau de satisfaction vis-à-vis du dispositif et leurs perceptions d’améliorations. De plus, une enquête qualitative a été réalisée auprès de ménages participants sous forme d’entretiens individuels semi-directifs réalisés en face-à-face et selon un guide d’entretien, afin d’explorer l’adéquation aux besoins et les améliorations perçues. L’échantillonnage des personnes interrogées dans le cadre de l’enquête qualitative s’est fait de manière à constituer un échantillon diversifiant les profils des enquêtés en fonction de la taille du ménage et de la modalité de transfert monétaire (carte Cohésia ou Nickel). Les entretiens, d’une durée comprise entre 1h30 et 2h30, ont été réalisés dans des centres sociaux de quartier, puis retranscrits.

### Evaluation de la faisabilité du processus de collecte de données pour le suivi d’indicateurs de sécurité alimentaire et bien-être

Le processus de collecte de données basé sur des questionnaires administrés en face à face avant (t0), trois mois (t1) et six mois (t2) après l’entrée dans le dispositif a été testé pour informer l'élaboration d'un futur essai randomisé contrôlé pour mesurer l'efficacité du dispositif. Les questionnaires comprenaient des questions relatives aux caractéristiques sociodémographiques, à la sécurité alimentaire et au bien-être des ménages, et à leur satisfaction vis-à-vis du dispositif.

Différents indicateurs relatifs à la sécurité alimentaire des ménages ont été testés: l’indicateur de suffisance alimentaire (Radimer, 2002) permettant de catégoriser les ménages en situation d’insuffisance alimentaire qualitative ou quantitative pour raisons financières; l’indice domestique de la faim (IDF) (Ballard, 2011) permettant de catégoriser les ménages en situation de faim modérée ou sévère; l’indice de diversité alimentaire minimale pour les femmes (Minimum Dietary Diversity For Women – MDDW); l’indice réduit des stratégies d’adaptation en réponse à l’insécurité alimentaire (Reduced Coping Strategy Index—rCSI); le nombre de repas par jour; et le recours à l’aide alimentaire (les données des 3 derniers indicateurs cités ont été collectées uniquement pour la phase 2).

Différents indicateurs de bien-être des participants ont été testés: l’indice de bien-être en cinq items de l’OMS (Topp et al., 2015), un score inférieur à 50 étant utilisé comme seuil de dépistage des personnes à risque de dépression; une échelle visuelle de détresse, et une échelle visuelle de perception du soutien social. Pour ces 2 échelles, il était demandé aux personnes de se situer, à l’aide de supports visuels, sur un gradient allant de 1 à 10. Pour la première échelle, un score supérieur à 6 est considéré comme signe de détresse, et pour la seconde un score inférieur ou égal à 4 est considéré comme indicateur de peu de soutien social.

La faisabilité du processus de collecte de données dans la population ciblée a été évaluée à partir des taux de participation aux différents temps d’enquête et les taux de réponses aux différents indicateurs. De plus, cette étude de faisabilité a permis d’évaluer le niveau d’insécurité alimentaire dans la population ciblée afin de pouvoir estimer la taille de l'échantillon nécessaire pour un futur essai contrôlé randomisé, et d’autre part de tester la réactivité ou sensibilité au changement des indicateurs de sécurité alimentaire et de bien-être après 3 mois.

### Analyses statistiques

Des analyses descriptives ont été réalisées à partir des réponses aux questionnaires pour renseigner la faisabilité du dispositif et du processus de collecte de données pour le suivi des indicateurs (taux de participation, taux de réponse aux indicateurs, caractéristiques socio-économiques des ménages, niveaux des indicateurs de sécurité alimentaire et de bien-être). Une analyse de puissance sur l’indicateur de suffisance alimentaire au niveau alpha 0,05 et au niveau bêta 0,80 a été réalisée pour estimer la taille de l'échantillon nécessaire pour un futur essai contrôlé randomisé.

L’analyse des entretiens qualitatifs a été réalisée en trois temps, dans une approche dite compréhensive (Charmillot et Seferdjeli, 2002): i) analyse des trajectoires (biographiques, professionnelles), des pratiques (économiques et alimentaires), des contraintes et des stratégies budgétaires des membres des ménages rencontrés afin de mieux comprendre le contexte d’insertion du TM, ii) analyse des usages et des arbitrages réalisés au cours de la période à l’aune de ces contextes, iii) mise en perspective de l’expérience du TM par rapport à celle de l’aide alimentaire conventionnelle, dans une démarche comparative, permettant aussi d’aborder les déterminants du non-recours à l’aide alimentaire.

## Résultats

### Faisabilité du dispositif

#### Mise en œuvre du ciblage

Treize acteurs sociaux de proximité (centres sociaux, antennes de quartier, associations) de la ville de Montreuil ont participé à l’identification de ménages en situation de précarité et à leur orientation vers le dispositif « Passerelle». Au total, 200 ménages volontaires ont participé à l’expérimentation du dispositif (82 ménages lors de la phase 1, 118 ménages lors de la phase 2).

Les caractéristiques sociodémographiques des ménages participants sont décrites dans le Tableau 1. Près de 90% des ménages étaient en situation de pauvreté monétaire, 89% en situation de pauvreté en conditions de vie, et 56% en situation de grande pauvreté. Une part importante des ménages était sans revenus issus d’une activité salariale à temps plein ou partiel (63%), en situation de monoparentalité (37%), d’endettement (57%). Sur la seconde phase, 56% des participants consommaient moins de trois repas par jour, 56% avaient déjà eu recours à une forme d’aide alimentaire au cours de leur vie, mais seulement 29% au cours du mois précédent l’entrée dans le dispositif.

**Tableau 1:** Caractéristiques sociodémographiques des ménages participants

	Phase 1	Phase 2	Total
	*N* = 76	*N* = 110	*N* = 186
*Représentant.e du ménage*			
Femme	68 (89.5%)	93 (84.5%)	161 (86.6%)
Age	46.4 (13.6)	47.2 (12.7)	46.9 (13.0)
*Composition du ménage*			
Nombre de personnes	4.5 (3.0–5.0)	4.0 (3.0–6.0)	4.0 (3.0–5.0)
Nombre d’enfants	1.5 (0.0–3.0)	2.0 (1.0–3.0)	2.0 (0.0–3.0)
Famille monoparentale	38%	35%	37%
Personne seule	7%	9%	8%
*Situation financière du ménage:*			
Revenu (€/mois/unité de consommation)^1^	702.0 (209.9)	751.1 (307.2)	726.5 (263.2)
En situation de pauvreté monétaire^1^	61 (96.8%)	53 (84.1%)	114 (90.5%)
En situation de privation matérielle et sociale^2^		80 (88.9%)	
En situation de grande pauvreté^3^		32 (56.1%)	
Avec au moins un membre ayant un revenu issu d’une activité salariée	29 (38.2%)	40 (36.4%)	69 (37.1%)
En situation d’endettement	45 (59.2%)	61 (55.5%)	106 (57.0%)
*Recours à l’aide alimentaire*			
Recours à une aide alimentaire au moins 1 fois au cours de la vie		62 (56.4%)	
Recours à une aide alimentaire au moins 1 fois au cours du dernier mois		18 (29.0%)	

Moyenne (E.T.) ou médiane (Q1-Q3) pour les variables continues, et n (%) pour les variables catégorielles. ^1^Données disponibles pour 63 ménages par phase, et 126 ménages au total. ^2^Données disponibles pour 90 ménages. ^3^Données disponibles pour 57 ménages

#### Mise en œuvre des 2 volets du dispositif

Le TM a été distribué sous forme de carte auprès de 194 ménages (n = 95 ménages sous forme de carte Nickel, et n = 99 sous forme de carte Cohésia), soit 97% de faisabilité. Seul 6 ménages n’avaient pas de pièce d’identité valide permettant l’attribution d’une carte de paiement; le TM leur a donc été fourni sous forme de chèques services.

Pour le volet orientation sociale, 199 ménages sur 200 ont été rencontrés par les coordinateurs.rices sociaux.ales pour réaliser le diagnostic social individualisé et les informer de leurs droits sociaux.

#### Acceptabilité par les usagers

L’enquête quantitative a montré que plus de 95% des ménages participants déclaraient être satisfait ou très satisfait du TM, sans différence significative selon le type de carte reçue. En ce qui concerne les perceptions d’amélioration, 69%, 50% et 45% des ménages ont respectivement déclaré avoir connu une amélioration en matière de quantité, de qualité, ou de diversité de leurs achats alimentaires grâce au dispositif.

L’enquête qualitative auprès des participants a permis de mieux comprendre l’acceptabilité du dispositif par les usagers. Au total 8 femmes ont participé à cette enquête, dont 5 recevant la carte Cohésia, 3 la carte Nickel, et dont la taille du ménage variait de 2 à 7 personnes. Les participantes ont déclaré apprécier la temporalité du versement du TM qui, effectué en milieu de mois, venait prendre le relai du compte-courant des ménages qui tendait alors à s’épuiser; la facilité d’usage des cartes; et la possibilité de les utiliser dans la plupart des enseignes auxquelles elles étaient habituées. Les participantes ayant eu à un moment de leur vie recours à l’aide alimentaire conventionnelle par des associations évoquaient apprécier la discrétion des cartes de paiement, qu’elles opposaient à la mise en visibilité lors du recours à l’aide alimentaire associative. Elles appréciaient la diversité et le caractère informel des critères d’éligibilité au dispositif; certaines n’ayant pas recours à l’aide alimentaire parce qu’elles appréhendaient de ne correspondre—où ne correspondaient effectivement pas—aux critères de revenu généralement retenus par les réseaux d’aides classiques, ou parce que leur demande auprès des circuits avait été éconduite auparavant. Le caractère partiellement restrictif de la carte Cohésia à certaines enseignes ne semblait pas avoir été vécu comme une contrainte; les participantes concernées témoignaient basculer au maximum leurs dépenses alimentaires sur cette carte et pouvoir réaliser de nouvelles dépenses (dépenses locatives, remboursement de dette, achats de vêtements, pratiques culturelles…) à partir de leur compte courant, en fonction de leurs besoins. La diversité des lieux d’achats autorisés semblait appréciée. Les participantes ayant reçu une carte Nickel témoignaient avoir apprécié de pouvoir réaliser des retraits en espèce leur permettant de conserver une organisation des dépenses proche de leurs habitudes, et facilitant la répartition du TM au sein du foyer (répartition conjugale, argent de poche pour les enfants, etc.) et le suivi des dépenses.

L’enquête qualitative a permis d’explorer dans quelle mesure le dispositif répondait aux besoins des usagers. Les informations recueillies suggéraient que le TM venait atténuer et prévenir des situations de privations alimentaires qui tendent d’ordinaire à s’accentuer au cours du mois avec l’épuisement du reste à vivre des ménages, entraînant une dégradation chronique du régime en matière de diversité, de qualité et parfois même de quantités consommées. En ce sens, le TM permettait –a minima- un « lissage» de la diversité et de la qualité de l’alimentation des ménages sur la période. Les entretiens suggéraient une hausse de la consommation de viande et de fruits, qui étaient les groupes alimentaires vis-à-vis desquels les enquêtées témoignaient le plus avoir modifié leur comportement en matière de fréquence de consommation, de quantité et de qualité des produits consommés.

***« ****La viande on mange un peu. On l'achète à la boucherie...Juste de temps en temps. Mais là, avec l'aide, j'ai fait plaisir à mes enfants. On est allé à la boucherie ensemble et j’ai pu leur acheter vraiment la viande qu'ils voulaient ».* (Kim.^1^ 48 ans, travail occasionnel, cinq enfants à charge)

***« ****Oui, ça m'a permis de prendre des fruits, plus de fruits, de se faire un peu plaisir. Après aussi c'est rapport à la santé.* (Ayoune, 33 ans, sans activité, deux enfants à charge)

***« ****Par exemple il y avait la fraise elle était vraiment chère. Ce que je peux goûter et que j'aime trop c'est les cerises. Là j’en ai pris un tout petit peu mais…à 9 euros le kilo ça c'est pas possible. Je ne peux pas faire des folies, il faut rester sage quand même. La fraise après, ça commençait, le prix à être dans la norme, j’en ai acheté. Après la banane, kiwi mais pas trop trop parce que ça reste cher aussi. Voilà c'est ça ».* (Caren, 56 ans, sans activité, trois enfants à charge)

Au cours des entretiens, les participantes déclaraient avoir augmenté la fréquence de leur repas, particulièrement à midi où elles avaient tendance—en l’absence du reste de la famille—à se contenter de grignoter, faute de moyens et d’énergie pour cuisiner des repas adaptés à leur envie. Elles témoignaient avoir moins eu recours à des stratégies de rationnement visant à favoriser l’alimentation de leur enfant au détriment de la leur, comme l’illustrent les verbatim ci-dessous.

« *Souvent d’habitude, ce que je fais à manger je lui donne [au bébé], il mange avec nous, et après je vais manger chez ma mère ».* (Merina, 27 ans, sans activité, un enfant à charge, enceinte)

« *Avant la nourriture ça nous suffisait pas. Souvent moi j'étais obligée... Enfin je préfère que mes enfants mangent après, et s’il y a des restes moi je mange* ». (Leila, 29 ans, sans activité, cinq enfants à charge)

Au-delà des dépenses pour l’alimentation à domicile, les participantes rencontrées ont déclaré s’être saisies du TM comme d’une occasion pour rembourser des dettes contractées auprès d’institutions (dettes locatives, découvert bancaire, amendes), auprès de la famille hors-foyer ou du voisinage (dettes alimentaires, vacances…). Elles déclaraient réaliser des dépenses orientées vers la satisfaction des besoins des enfants: vêtement, restauration hors-domicile, pratiques culturelles (cinéma, sport), argent de poche, etc. Les participantes déclaraient aussi avoir pu réaliser des dépenses de bien-être (vêtement, coiffeur, sorties entre amies, …) qui passent d’ordinaire en bas des priorités des dépenses des ménages et sont systématiquement sacrifiées. L’enquête qualitative semblait aussi confirmer que les décisions associées aux usages des cartes étaient essentiellement prises en charge par les femmes des ménages. Pour plusieurs d’entre elles, leur mari n’était pas au courant du TM associé au dispositif.

*«* *Des fois on a envie de faire plaisir aux enfants, de les amener au Mcdo, au Quick, au cinéma...Mais ça moi j'ai pas ça, je peux pas me le permettre ».* (Amel, 63 ans, situation de handicap, trois enfants à charge)

***« ****Quand j'ai reçu la carte, je me suis autorisée à me payer le coiffeur. Ça faisait dix ans que je n’y étais pas allée, je pouvais pas me le permettre… Pareil pour les habits, ça faisait vraiment longtemps. Vous voyez j'ai des vêtements ils ont 15 ans* ». (Jeanne, 51 ans, travail en temps-partiel, un enfant au foyer)

L’alimentation hors domicile fait partie des dépenses mises en suspens suite à la crise du COVID19 ou une perte de revenu, que les enquêtées déclaraient avoir pu réinvestir (essentiellement en famille, avec leur enfants) grâce au TM. Des commentaires libres déclarés par les participants au cours de l’enquête par questionnaire précisaient qu’une partie d’entre eux avait profité du TM pour réaliser des achats d’électroménager (frigo, micro-onde, plaque chauffante, mixeur etc.) ou de mobilier (canapé, lit, housse etc.).

### Faisabilité du processus de collecte de données

Les taux de participation aux enquêtes t0, t1 et t2 étaient de 93% (*n* = 186), 76% (*n* = 142) et 73% (*n* = 135), respectivement. Les taux de participation étaient similaires lors des deux phases de déploiement. Dans l’échantillon final, les taux de non-réponses concernant les indicateurs de sécurité alimentaire et de bien-être étaient inférieurs à 5% (Tableau 2), excepté pour le MDDW (jusqu’à 20% de non-réponse).

**Tableau 2:** Taux de réponse par indicateur et par temps de suivi

	t0 *n* = 186	t1 *n* = 142	t2 *n* = 135
Suffisance Alimentaire	180 (97%)	142 (100%)	132 (98%)
Indice domestique de la faim	186 (100%)	142 (100%)	135 (100%)
MDD-W	149 (80%)	126 (89%)	122 (90%)
r-CSI (phase2)	106/110 (96%)	79/79 (100%)	81/82 (99%)
Score de bien-être	178 (96%)	138 (97%)	128 (95%)
Echelle de perception de soutien social	179 (96%)	137 (96%)	131 (97%)
Echelle de détresse	181 (97%)	138 (97%)	129 (96%)

Les niveaux des indicateurs de sécurité alimentaire et de bien-être dans l’échantillon avant d’entrer dans le dispositif (t0) et après 3 mois (t1) et 6mois (t2) sont présentés dans le Tableau 3.

**Tableau 3:** Niveaux des indicateurs de sécurité alimentaire et de bien-être des ménages avant (t0), après 3 mois (t1) et après 6 mois (t2) de réception du dispositif

	t0	t1	t2
	*n* = 186	*n* = 142	*n* = 135
Indicateur de suffisance Alimentaire: proportion de ménages en situation de suffisance alimentaire (IC95)	17,7% (12,4%−24,0%)	35,2% (27,4% – 43,7%)	20,5% (13,9%−28,3%)
Indice domestique de la faim: proportion de ménages en situation de peu ou pas de faim (IC95)	75,3% (68,4%−81,3%)	89,4% (83,2%—94,0%)	85,2% (78,1%—90,7%)
MDD-W: proportion de ménages au-dessus du seuil de diversité alimentaire (IC95)	50,3% (42,0%—58,6%)	65,1% (56,1%—73,4%)	49,2% (40,0%—58,4%)
r-CSI: score moyen (IC95)	13,6 (11,3–16,0)	8,6 (6,2–11,0)	14,4 (11,6–17,3)
Indice de bien-être: proportion de ménages au-dessus du seuil de bien-être (IC95)	50,0% (42,4%−57,6%)	65,2% (56,6%−73,1%)	61,0% (51,9%—69,4%)
Echelle de perception de soutien social: proportion de ménages au-dessus du seuil de soutien social (IC95)	53,1% (45,5%—60,1%)	67,2% (58,6%—74,9%)	63,4% (54,5%—71,6%)
Echelle de détresse: proportion de ménages en dessous du seuil de détresse (IC95)	48,1% (40,6%—55,6%)	81,9% (74,4%—87,9%)	69,0% (60,3%—76,8%)

A t0, la part des ménages en situation de suffisance alimentaire était de 17.7%. Cette estimation et le taux d’attrition à 3 mois d’évaluation ont servi de base pour estimer la taille d’échantillon du futur essai randomisé contrôlé: sous l’hypothèse d’un coefficient de corrélation intraclasse de 0,02, un échantillon de 370 ménages permettra de mesurer l’impact de l’intervention avec une puissance statistique supérieure à 80% et une erreur de type I de 5%.

En ce qui concerne la réactivité des indicateurs de sécurité alimentaire: après 3 mois (t1) la proportion des ménages en situation de suffisance alimentaire avait doublé (+ 99%), celle des ménages en situation de peu ou pas de faim avait augmenté de + 19%, celle des ménages atteignant le seuil minimal de diversité alimentaire de + 29%, et le score moyen de recours à des stratégies d’adaptation alimentaire avait baissé de 37% par rapport à leurs niveaux à t0.

Pour la réactivité des indicateurs de bien-être: à t1, les proportions de participants au-dessus des seuils de bien-être, de soutien social, et en dessous du seuil de détresse avaient respectivement augmenté de 30%, 27% et 70% par rapport à leur niveau à t0.

## Discussion

En accord avec les recommandations du MRC pour le développement d'interventions complexes (Richards et Hallberg, 2015; Skivington et al., 2021), cette étude a exploré la faisabilité d’un nouveau dispositif de lutte contre la précarité alimentaire en France proposant pour la première fois un TM non fléché couplé à une orientation sociale (le dispositif « Passerelle») afin de déterminer l’intérêt et préparer la conception d’un futur essai randomisé contrôlé pour évaluer son efficacité. Les résultats montrent la faisabilité de mise en œuvre des 2 volets du dispositif (TM et orientation sociale) et du ciblage des ménages en situation de précarité par les acteurs sociaux de proximité, ainsi que l’acceptabilité rétrospective du dispositif par les usagers. L’étude a également montré la faisabilité du processus de collecte de données auprès de la population ciblée pour le suivi d’indicateurs de sécurité alimentaire et de bien-être, et fourni les données préliminaires nécessaires à la conception du futur essai.

### Faisabilité de mise en œuvre et acceptabilité du dispositif

Le dispositif « Passerelle» s’appuie sur les acteurs sociaux de proximité (centres sociaux, antennes de quartier, associations) pour identifier les ménages en situation de précarité à qui proposer le dispositif, sans demander de justificatif de ressources. Cette étude a montré que ce processus d’identification des ménages par les acteurs sociaux a permis de cibler des ménages très précaires en situation de logement ordinaire sur le territoire: près de 90% des ménages participants étaient en situation de pauvreté monétaire et 89% en situation de privation matérielle et sociale (pauvreté en conditions de vie) (contre 14% et 13% en population générale (INSEE, 2024)). Plus spécifiquement, le processus de ciblage a permis d’atteindre des ménages en situation de précarité alimentaire: plus de 80% des ménages étaient en situation d’insuffisance alimentaire pour raisons financières, dont 33% d’insuffisance alimentaire quantitative (20% en population générale, dont 3% d’insuffisance alimentaire quantitative (AnSES, 2017)). Ces prévalences sont proches de celles des publics du SNAP (Supplemental Nutrition Assistance Program) aux Etats-Unis (65% en insécurité alimentaire (Mabli et Ohls, 2015)) et l’intensité des privations et du recours à des stratégies d’adaptation se rapproche de ceux des publics de l’aide alimentaire en France (Accardo et al., 2022a). Enfin, le ciblage a permis d’atteindre des publics en situation de non recours à l’aide alimentaire conventionnelle: bien qu’en situation d’insécurité alimentaire, seul 29% des participants avait eu recours à l’aide alimentaire au cours du mois précédent l’entrée dans le dispositif.

Cette étude a permis de tester la mise en œuvre des 2 volets du dispositif et d’explorer son acceptabilité par les usagers. Le nombre de participants (200 ménages volontaires), les taux très élevés de distribution des cartes de TM, de participation aux rendez-vous d’orientation sociale (tous deux proches de 100%) et de satisfaction (95%) attestent de la faisabilité opérationnelle du dispositif et reflètent la forte adhésion des usagers à ce dernier. L’enquête qualitative a suggéré que le format (carte de paiement) et le caractère non restrictif (carte Nickel) ou peu restrictif (carte Cohésia) du TM qui permettait de réaliser des achats alimentaires et autres qu’alimentaires a participé à renforcer son acceptabilité. Le format carte de paiement offrait une facilité d’appropriation et une discrétion susceptible de minimiser les effets de stigmatisation et donc le taux de non-recours, identifié par la littérature comme un problème central des dispositifs utilisant des chèques alimentaires comme support de TM (Panzera et al., 2017; Vasan et al., 2021), des TM restrictifs (Johnson, 2015), ou bien de l’aide alimentaire (Bonzi, 2019; Caillavet et al., 2021) dans les pays à revenu élevé**.** Dans le cadre du dispositif WIC (Special Supplemental Nutrition Program for Women, Infants, and Children) aux Etats-Unis, le passage du chèque alimentaire à la carte de paiement a d’ailleurs accru le taux de recours de presque 8 points en trois ans (Vasan et al., 2021). Comme observé dans d’autres études (Brocard et Saujot, 2022; Poblacion et al., 2017), l’enquête qualitative a montré des pratiques de substitution d’achats faits à partir du compte-courant et avec la carte du TM lorsque ce dernier était partiellement restreint à certains types d’enseigne (carte Cohésia). Ces résultats questionnent la pertinence d’un fléchage des TM, par exemple vers l’alimentation, puisque le fléchage peut par ailleurs induire une logistique et un paramétrage plus contraignant pour la mise en œuvre, et réduire la facilité d’usage et la satisfaction des usagers.

Les enquêtes quantitative et qualitative ont permis d’explorer l’efficacité perçue par les participants, dont la majorité déclarait pouvoir améliorer leurs achats alimentaires, avec des améliorations portant à la fois sur la quantité, la qualité, et la diversité des aliments consommés. L’enquête qualitative a notamment permis de préciser que ces améliorations perçues semblaient essentiellement portées par la hausse de la fréquence de consommation, des quantités consommées, et de la qualité de certains groupes alimentaires (viandes, fruits, et dans une moindre mesure produits laitiers et légumes), ainsi que par l’augmentation de la restauration hors-domicile. Dans un contexte de transition nécessaire vers des régimes alimentaires sains et durables (FAO & WHO, 2019), les changements de pratiques évoqués dans l’enquête qualitative ont suggéré l’intérêt d’ajouter une composante à l’intervention qui viserait à sensibiliser les usagers quant aux bénéfices sur la santé et l’environnement d’une consommation modérée de produits d’origine animale (viande rouge notamment) et riche en fruits & légumes, et à échanger collectivement sur des pratiques favorisant cet équilibre.

L’enquête qualitative a aussi permis d’explorer dans quelle mesure le dispositif répondait aux besoins des ménages et de faire des hypothèses sur les mécanismes pouvant concourir à l’amélioration de leur bien-être. Le plus observé dans cette enquête concerne l’effet du désendettement sur l’allégement de la charge mentale des ménages, notamment dans la mesure où celui-ci leur assure la possibilité de recourir à nouveau à l’endettement à l’avenir si nécessaire. Vient ensuite la réalisation de dépenses de consommation courantes depuis longtemps retardées, permettant de répondre à une partie des besoins des enfants (habillement, pratiques culturelle, de sorties, de sociabilité etc.) ainsi qu’à ceux des mères (vêtements, bien-être), d’ordinaire systématiquement relégués au dernier plan des priorités des ménages et sacrifiés. L’achat de biens électroménagers ou de mobilier venait aussi répondre à des besoins de plus long terme. Enfin, les orientations sociales proposées dans le cadre du dispositif ont débouché pour certains participants sur un accompagnement effectif (accès gratuit à un suivi psychologique, échelonnement de dettes, aide administrative, formation recherche-emploi) contribuant à l’amélioration à plus long terme de leur bien-être.

### Faisabilité du processus de collecte de données pour le suivi d’indicateurs

Plusieurs résultats corroborent la faisabilité du processus de collecte de données pour le futur essai randomisé: un taux de participation de plus de 90% lors de la première enquête par questionnaire, des taux d’attrition à 3 mois et à 6 mois inférieurs à 30%, cohérents avec ceux généralement observés en recherche interventionnelle en santé des populations, et des taux élevés de complétion des questions relatives aux indicateurs de suivi. De plus, l’étude a permis d’estimer la proportion de ménages en situation d’insuffisance alimentaire dans la population ciblée et calculer la taille d’échantillon nécessaire au futur essai pour l’évaluation d’impact du dispositif. Enfin, l’étude a montré la pertinence des indicateurs de suivi. D’une part l’amélioration importante des différents indicateurs de sécurité alimentaire et de bien-être à t1 (de 30 à près de 100% de variation, excepté pour l’indice de la faim) atteste de leur sensibilité au changement après 3 mois. Plus spécifiquement, l’étude suggère une forte réactivité de l’indicateur de suffisance alimentaire dont le niveau a doublé après 3 mois, ce qui conforte l’utilisation de cet indicateur dans le cadre du futur essai d’efficacité. A l’inverse, l’indice de la faim ayant montré la plus faible variation (+ 19%) n’est pas à privilégier. D’autre part l’observation des évolutions et des amplitudes de changement similaires sur les deux phases de déploiement du dispositif, réalisées à 4 mois d’écart, au cours de saisons et dans de zones d’intervention différentes, atteste de la robustesse des indicateurs.

### Limites de l’étude

Cette étude présente des limites. L’expérimentation ne comportant pas de processus de randomisation et de groupe de contrôle, elle ne permet pas de tester l’acceptabilité de ce design par les participants. De plus, le dispositif a été testé dans un seul contexte (ville de Montreuil), ce qui peut soulever des questions quant à sa faisabilité et son acceptabilité dans d'autres contextes. L’évaluation de l’acceptabilité réalisée de manière rétrospective pour explorer la satisfaction des usagers n’a permis d’aborder qu’une partie des composantes de l’acceptabilité telles que définie par Sekhon et al*.* (Sekhon et al., 2017), notamment le ressenti des participants vis-à-vis du dispositif, sa facilité d’utilisation, ou l’efficacité perçue; l’enquête aurait gagné à explorer plus spécifiquement d’autres composantes telles que l’éthique (e.g. dans quelle mesure le dispositif semble juste et équitable) ou la cohérence de l’intervention du point de vue des usagers. Enfin, l’intégralité des personnes rencontrées dans le cadre de l’enquête qualitative étaient des femmes, ce qui peut constituer un biais mais celui-ci reste limité dans la mesure où les femmes représentaient une très large majorité (92%) des participants au dispositif.

Comme recommandé par le cadre du MRC, cette étude aurait gagné à formaliser dans un premier temps la théorie du programme, afin d’expliciter en amont les modalités d’articulation et d’interaction des différentes composantes de l’intervention, leurs mécanismes, les changements attendus, ou encore les caractéristiques du contexte susceptibles d’influencer ces mécanismes.

## Conclusion et perspectives

Cette étude a montré la faisabilité du nouveau dispositif « Passerelle» de lutte contre la précarité alimentaire en France, en matière de mise en œuvre du ciblage par les acteurs sociaux du territoire pour atteindre des ménages en situation de précarité et de non-recours, de mise en œuvre des 2 volets du dispositif (TM non fléché et orientation sociale), et d’acceptabilité par les usagers après expérimentation du dispositif. Elle a également montré la faisabilité du processus de collecte de données auprès de ménages en situation de précarité pour le suivi d’indicateurs de sécurité alimentaire et de bien-être. Par conséquent, il apparait pertinent de poursuivre cette étude de faisabilité par une évaluation d’efficacité du dispositif « Passerelle». Les enseignements de cette étude serviront à construire un essai randomisé contrôlé pour mesurer les effets sur la sécurité alimentaire des ménages attribuables au dispositif, en vue d’enrichir les connaissances sur les dispositifs de TM et d’alimenter les réflexions sur les politiques de lutte contre la précarité alimentaire et de réduction des inégalités sociales de santé liées à l’alimentation en France.

## Contributions à la connaissance

Qu’est-ce que cette étude ajoute aux connaissances existantes?

- Faisabilité de mise en œuvre des 2 volets (transfert monétaire et orientation sociale) du dispositif « Passerelle»
- Faisabilité du processus de ciblage des ménages en situation de précarité par une approche d’aller-vers mobilisant les acteurs sociaux de proximité
- Acceptabilité du dispositif « Passerelle» par les usagers
- Faisabilité du processus de collecte de données pour le suivi d’indicateurs de sécurité alimentaire et de bien-être dans la population ciblée

Quelles sont les principales conséquences qu’il y a lieu d’en tirer pour les interventions, la pratique ou les politiques en santé publique?

- Pertinence de poursuivre par une étude d’efficacité du dispositif « Passerelle» dans un essai randomisé contrôlé
- Sensibilité au changement de l’indicateur de suffisance alimentaire
- Sur la base de la proportion de ménages en situation d’insuffisance alimentaire dans la population cible, un échantillon de 370 ménages est nécessaire pour un futur essai randomisé contrôlé
- Faisabilité opérationnelle et acceptabilité par les usagers d’un dispositif de transfert monétaire non fléché en France

## Supplementary Information

Below is the link to the electronic supplementary material.

ESM 1 (DOCX 17.6 KB)

## Data Availability

A l’exception des données de l’enquête qualitative qui ne peuvent pas être rendues publiques en raison de restrictions éthiques et de confidentialité, les données de l’étude sont disponibles auprès de l’auteur correspondant, sur demande raisonnable.

## Footnotes

1 Dans le respect de la confidentialité des témoignages, les pré*noms des personnes* interrogées ont été *modifiés.*

## Disponibilité du code

NA

## Déclarations

### Approbation éthique

L’étude a été menée dans le respect des principes de la déclaration d’Helsinki et du règlement général européen sur la protection des données (RGPD). Le protocole de l’étude a obtenu un avis favorable du Comité d’Evaluation Ethique de l’Inserm (IRB00003888, avis n°22–890).

### Consentement à participer

Tous les participants ont donné leur consentement écrit pour participer, après avoir été informés du protocole d’étude.

### Consentement à la publication

NA

### Conflit d'intérêts

Aucun conflit d’intérêts à déclarer.
