# Supplementary material for: Étude de faisabilité du dispositif « Passerelle » couplant transfert monétaire non fléché et orientation sociale destiné à des ménages en situation de précarité en France
Source: Can J Public Health. 2026 Apr 29;117(Suppl 1):104–16. doi: 10.17269/s41997-025-01120-7 (PMC13129010; doi:10.17269/s41997-025-01120-7)
Supplement: Supplementary file 1 — (DOCX 17.6 KB) [file 41997_2025_1120_MOESM1_ESM.docx]

**Documents électroniques supplémentaires**

**Titre de l’article** : Étude de faisabilité du dispositif « Passerelle » couplant transfert monétaire non fléché et orientation sociale destiné à des ménages en situation de précarité en France

**Journal** : Canadian Journal of Public Health

**Tableau Supplémentaire 1 : Description de l’intervention (dispositif « Passerelle ») selon les items de la grille TIDieR-PHP** (Campbell et al., 2018)

| **Item de la grille TIDieR-PHP** | **Description de l’intervention** |
| --- | --- |
| 1 Brief name | Dispositif "Passerelle" |
| 2 Why | Le dispositif « Passerelle » est un nouveau dispositif de lutte contre la précarité alimentaire proposé par 2 associations en France : Action Contre la Faim et la Fondation de l’Armée du Salut. L’objectif du dispositif est de réduire les inégalités sociales liées à l’alimentation en agissant sur les capacités économiques des ménages en situation de précarité et leur accès aux droits sociaux. |
| 3 What materials | Le dispositif comprend 2 volets : un transfert monétaire (TM) non fléché et une orientation sociale individualisée.  Volet TM : Deux types de carte ont été expérimentés pour la mise à disposition du TM : i) carte « Nickel », utilisable dans tous types de magasins et permettant aussi le retrait d’espèces et les paiements en ligne, et ii) carte « Cohésia », utilisable dans toutes les enseignes d’alimentation (supermarché, restaurant, boulangerie, boucherie, épicerie,…), de santé (pharmacie, médecin, hôpital), d’énergie (station-essence), de transports (taxi, bus, métro, train) et de services (blanchisserie, poste, impôts). Lorsque les pièces d’identité nécessaires à la création des cartes n’étaient pas transmises à temps ou n’étaient pas valides, le TM a été distribué sous forme de chèques services.  Volet orientation sociale : un travail de cartographie des dispositifs et de rencontres des acteurs de l’action sociale présents sur le territoire montreuillois a été réalisé par les coordinateur.rices sociaux.ales en amont des rendez-vous afin de proposer des orientations adéquates. Un outil de diagnostic social a constitué le support des entretiens sociaux pour échanger autour de la situation globale de la personne, et des membres de sa famille. |
|  |  |
|  |  |
|  |  |
| 4 What and how | Le dispositif comprend 2 volets : un TM non fléché et une orientation sociale individualisée.  Le volet TM consiste consistait à distribuer une allocation non fléchée de 63€/mois par personne du ménage pendant 4 mois sous forme de carte de paiement. Ce montant a été défini sur la base du coût minimum nécessaire pour respecter l’ensemble des recommandations nutritionnelles (estimé à 3,85 €/j) (Maillot et al., 2017) et avec l’intention de couvrir les besoins au-delà du strict minimum alimentaire (e.g. transport) et de prendre en compte l’inflation observée en 2022, soit un montant réévalué à 4,5€/jour/personne, pour deux semaines par mois. Deux types de carte ont été expérimentés pour la mise à disposition du TM : i) carte « Nickel », utilisable dans tous types de magasins et permettant aussi le retrait d’espèces et les paiements en ligne, et ii) carte « Cohésia », utilisable dans toutes les enseignes d’alimentation (supermarché, restaurant, boulangerie, boucherie, épicerie,…), de santé (pharmacie, médecin, hôpital), d’énergie (station-essence), de transports (taxi, bus, métro, train) et de services (blanchisserie, poste, impôts). Lorsque les pièces d’identité nécessaires à la création des cartes n’étaient pas transmises à temps ou n’étaient pas valides, le TM a été distribué sous forme de chèques services.  Le volet « orientation sociale » du dispositif consistait à proposer aux participants un rendez-vous avec un travailleur social afin d’établir un diagnostic social individualisé. En fonction du diagnostic, les ménages étaient alors orientés vers des structures associatives présentes sur le territoire (épicerie solidaire, association d’accompagnement psychologique gratuit, écrivain public, etc) ou de l’accès aux droits (intermédiation d’échelonnement de dette auprès de l’OPHM, formations via Pole Emploi, accès à des chantiers d’insertion etc.). |
|  |  |
|  |  |
|  |  |
|  |  |
|  |  |
| 5 Who provided | Le volet TM (distribution de la carte et réalisation des versements mensuels) a été mis en œuvre par le personnel d'Action Contre la Faim. Les rendez-vous d'orientation sociale ont été réalisés par des coordinateur.rices sociale de la Fondation de l'Armée du Salut |
| 6 Where | Le dispositif a été déployé dans des quartiers prioritaires de la ville de Montreuil, France |
| 7 When and how often | Le dispositif « Passerelle » a été déployée en 2022 dans cinq quartiers classés prioritaires de la ville (QPV) de Montreuil, France. Le déploiement du dispositif s’est organisé en deux phases de 4 mois : de janvier à avril, et de juin à septembre. |
| 8.1 Planned variation | Lorsque les pièces d’identité nécessaires à la création des cartes n’étaient pas transmises à temps ou n’étaient pas valides, le TM a été distribué sous forme de chèques services. |
| 8.2 Unplanned variation | NA |
| 9.1 How well | Volet TM : Un suivi téléphonique rapproché (avec un numéro et dédié) a été mis en place afin de permettre aux participants de remonter leurs interrogations quant à l’utilisation des cartes, aux dates des versements, ou bien signaler un problème ou un dysfonctionnement (carte inactive, code d'accès oublié, changement de numéro de téléphone de contact, etc.). Volet orientation sociale : les coordinateur.rices sociales ont renseigné un fichier de suivi des orientations permettant de suivre l’avancement des orientations avec les acteur·ices vers qui les personnes avait été orientées, et avec les personnes elles-mêmes (appels, envoi de sms, accompagnement/participation en physique au rdv…). |
| 9.2 How well—delivery | Le TM a été distribué sous forme de carte auprès de 194 ménages (n=95 ménages sous forme de carte Nickel, et n=99 sous forme de carte Cohésia) sur les 200 participants. Seul 6 ménages n’avaient pas de pièce d’identité valide permettant l’attribution d’une carte de paiement et le TM leur a donc été fourni sous forme de chèques services.  Sur le volet orientation sociale, 199 ménages sur 200 ont été rencontrés pour réaliser le diagnostic individualisé et les informer de leurs droits sociaux. |
